# Cytomegalovirus enteritis as an unusual cause of small bowel obstruction in an immunocompetent woman: a case report

**DOI:** 10.1093/jscr/rjag108

**Published:** 2026-02-26

**Authors:** José Antonio Vergara Torrente, Gerardo Muñoz Maldonado, Israel García Avilan, Carlos Pacheco Molina

**Affiliations:** Universidad Autonoma de Nuevo Leon, General Surgery, Monterrey, Nuevo león, México; Universidad Autonoma de Nuevo Leon, Head of the Deparment of General Surgery, Monterrey, Nuevo león, México; Universidad Autonoma de Nuevo Leon, General Surgery, Monterrey, Nuevo león, México; Universidad Autonoma de Nuevo Leon, General Surgery, Monterrey, Nuevo león, México

**Keywords:** cytomegalovirus, enteritis, intestinal obstruction, immunocompetent

## Abstract

A 57-year-old woman without comorbidities presented with severe abdominal pain, hematemesis, and melena. Contrast enhanced computed tomography demonstrated small bowel obstruction with two transition points in the proximal jejunum. Endoscopy revealed a circumferential jejunal ulcer larger than 5 cm with loss of vascular pattern, induration, whitish exudate, and contact bleeding. Segmental resection with side-to-side anastomosis was performed. Histopathology and immunohistochemistry confirmed cytomegalovirus infection. Intravenous ganciclovir was administered for 7 days, with a favorable clinical course and discharge in good condition. Cytomegalovirus should be considered in the differential diagnosis of acute intestinal obstruction even in the absence of immunosuppression. Early histologic confirmation and individualized treatment combining surgery and antiviral therapy are associated with favorable outcomes.

## Introduction

Cytomegalovirus (CMV), a ubiquitous herpesvirus with high global seroprevalence, can involve the gastrointestinal tract; however, small-bowel disease remains uncommon even across large gastrointestinal cohorts [1]. In immunocompetent adults, clinical and endoscopic presentations are frequently nonspecific ranging from abdominal pain and bleeding to ileus or frank obstruction so diagnostic certainty typically depends on tissue confirmation rather than clinical suspicion alone [2, 3]. Moreover, CMV biology latency with potential reactivation helps explain why overt disease may arise in the absence of recognized immunosuppression, yet without pathognomonic features at presentation [2, 3]. Given this overlap with common surgical and gastroenterological differentials, immunohistochemistry (IHC) on mucosal biopsies is pivotal to demonstrating tissue invasive infection and to distinguish clinically relevant disease from incidental or latent viral detection; polymerase chain reaction may be complementary but risks identifying latent infection without proving invasiveness [4]. Because most gastrointestinal CMV disease affects the colon and rectum, small-bowel manifestations may be underrecognized, contributing to delayed diagnosis and treatment [1]. We report a case of jejunal CMV enteritis presenting intestinal obstruction in an immunocompetent patient, highlighting the diagnostic value of targeted tissue sampling with IHC and outlining practical therapeutic considerations for a multidisciplinary approach.

## Case report

A previously healthy 57-year-old woman presented with severe abdominal pain, hematemesis, anorexia, and melena. On admission, vital signs were as follows: heart rate 90 beats per minute, respiratory rate 19 breaths per minute, oxygen saturation 95% on room air, and temperature 36 degrees Celsius. During hospitalization, she developed oral intake intolerance, vomiting, progressive abdominal distension, and worsening pain.

**Figure 1:**
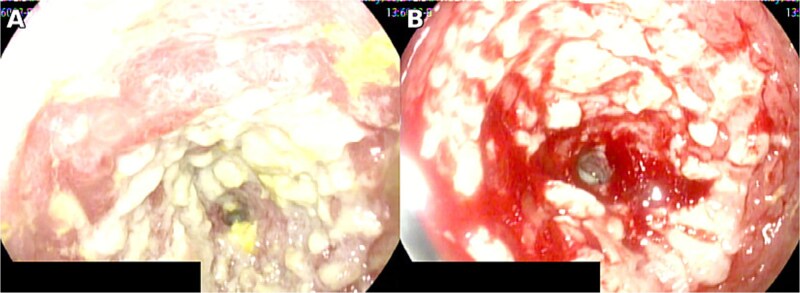
Circumferential jejunal ulcer measuring >5 cm, with loss of vascular pattern, induration, whitish exudate, and contact bleeding.

Initial laboratory testing showed leukocytosis of 12 000 per microliter with neutrophil predominance of 82%, creatinine 0.7 mg per deciliter, and liver function tests within normal limits. She had no relevant past medical history, denied corticosteroid use or other immunosuppressive therapy, and had a negative HIV test; therefore, she was considered immunocompetent. Contrast enhanced computed tomography demonstrated small bowel obstruction with two transition points in the proximal jejunum. Endoscopy revealed a circumferential jejunal ulcer larger than 5 cm with loss of vascular pattern, induration, whitish exudate, and contact bleeding (Fig. 1).

**Figure 2:**
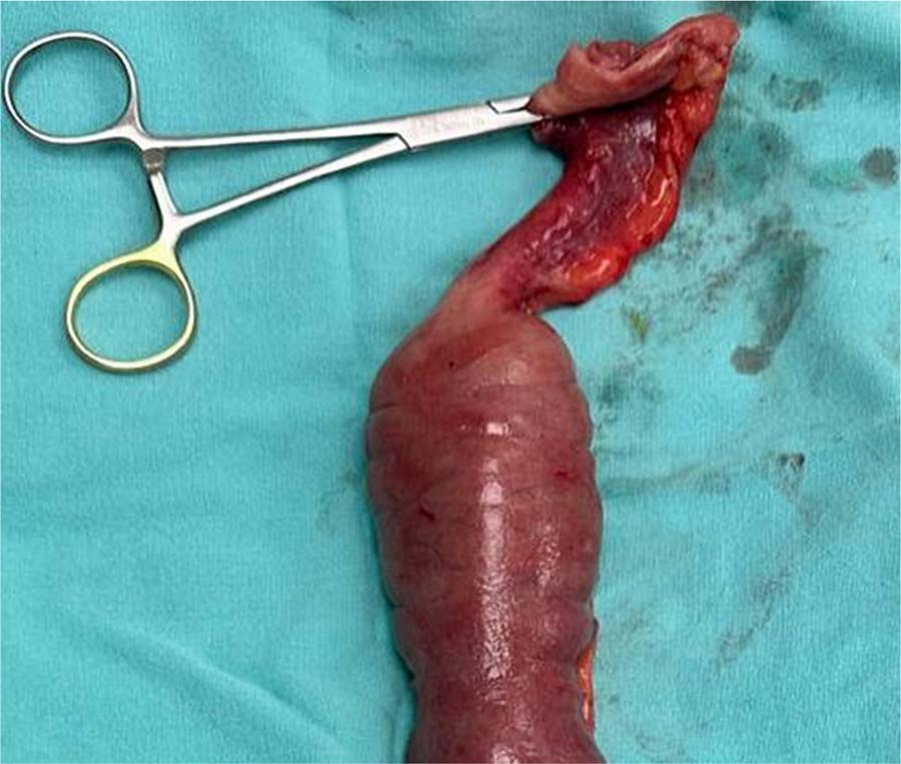
Stenotic thickened jejunal segment.

Laparotomy identified a stenotic, thickened segment ~50 cm distal to the ligament of Treitz (Fig. 2), and segmental resection with side-to-side anastomosis was performed. Histopathology and IHC confirmed CMV infection. Intravenous ganciclovir was initiated and continued for 7 days, with a favorable clinical course. At 1-week postoperative follow up, there were no signs of surgical site infection; the patient tolerated oral intake and had normal flatus and bowel movements. At 1-month and 3-month follow-up visits, she remained asymptomatic without new clinical abnormalities. Histopathological and IHC analyses confirmed CMV infection. Intravenous ganciclovir therapy was initiated with favorable outcomes [5, 6].

## Discussion

Jejunal involvement by CMV in immunocompetent adults is uncommon and typically presents with nonspecific features of intestinal obstruction [7, 8]. Although gastrointestinal CMV more often affects the colon and rectum, small-bowel disease remains relatively unusual even across large cohorts, which contributes to delayed recognition [1]. In 11 immunocompetent hosts, presentations are heterogeneous and risk factors are often absent or subtle; maintaining a high index of suspicion is therefore essential, particularly when symptoms evolve or fail to respond to conservative measures [2, 3].

Clinical manifestations overlap with other causes of acute abdomen or obstruction; hence, 17 tissue diagnosis is required, demonstrating cytomegalic inclusions and positive IHC [2–4, 9]. In practice, IHC on tissue provides high specificity, whereas PCR may be complementary but can detect latent infection without proving tissue-invasive disease [4]. When the small bowel is involved, device-assisted enteroscopy enables targeted biopsies beyond the reach of standard endoscopy, thereby improving diagnostic yield [9]. In our patient, an extensive circumferential jejunal ulcer with stenosis, CT signs of obstruction, and lack of clinical improvement justified intestinal resection with anastomosis. Endoscopic patterns reported in immunocompetent individuals include irregular or circumferential ulcers with exudate and diffuse erythema, consistent with our findings [5]. In addition, imaging that demonstrates fixed transition points with proximal dilatation correlates with the need for operative management when clinical deterioration persists despite supportive care [6]. Concordance between imaging and intra-operative findings supported surgical planning, and histological confirmation guided antiviral therapy.

Management should be individualized: surgery addresses mechanical complications, and antivirals are considered in severe disease or where there is tissue evidence of infection [2, 3, 10]. Although some immunocompetent patients improve without antivirals, contemporary reviews emphasize reserving ganciclovir/valganciclovir for complicated presentations or systemic compromise, balancing potential hematological and renal toxicities against expected benefit [2, 10]. In our case, tissue-proven infection together with 45 obstructions justified a combined approach, which is consistent with recent literature and was associated with a favorable outcome [2, 5, 6, 10].
